# Molecular mechanisms of human coronavirus NL63 infection and replication

**DOI:** 10.1016/j.virusres.2023.199078

**Published:** 2023-02-22

**Authors:** Gino Castillo, Juan Carlos Mora-Díaz, Mary Breuer, Pallavi Singh, Rahul K Nelli, Luis G Giménez-Lirola

**Affiliations:** a Department of Veterinary Diagnostic and Production Animal Medicine, Veterinary Diagnostic Laboratory, College of Veterinary Medicine, Iowa State University, 1850 Christensen Drive, Ames, IA 50011, USA; b Department of Biological Sciences, Northern Illinois University, DeKalb, IL 60115, USA

**Keywords:** Human coronavirus NL63, HCoV-NL63, Infection, Replication

## Abstract

Human coronavirus NL63 (HCoV-NL63) is spread globally, causing upper and lower respiratory tract infections mainly in young children. HCoV-NL63 shares a host receptor (ACE2) with severe acute respiratory syndrome coronavirus (SARS-CoV) and SARS-CoV-2 but, unlike them, HCoV-NL63 primarily develops into self-limiting mild to moderate respiratory disease. Although with different efficiency, both HCoV-NL63 and SARS-like CoVs infect ciliated respiratory cells using ACE2 as receptor for binding and cell entry. Working with SARS-like CoVs require access to BSL-3 facilities, while HCoV-NL63 research can be performed at BSL-2 laboratories. Thus, HCoV-NL63 could be used as a safer surrogate for comparative studies on receptor dynamics, infectivity and virus replication, disease mechanism, and potential therapeutic interventions against SARS-like CoVs. This prompted us to review the current knowledge on the infection mechanism and replication of HCoV-NL63. Specifically, after a brief overview on the taxonomy, genomic organization and virus structure, this review compiles the current HCoV-NL63-related research in virus entry and replication mechanism, including virus attachment, endocytosis, genome translation, and replication and transcription. Furthermore, we reviewed cumulative knowledge on the susceptibility of different cells to HCoV-NL63 infection *in vitro*, which is essential for successful virus isolation and propagation, and contribute to address different scientific questions from basic science to the development and assessment of diagnostic tools, and antiviral therapies. Finally, we discussed different antiviral strategies that have been explored to suppress replication of HCoV-NL63, and other related human coronaviruses, by either targeting the virus or enhancing host antiviral mechanisms.

## Highlights

• HCoV-NL63 being the most prevalent human coronaviruses after HCoV-OC43.
• HCoV-NL63 cause upper and lower respiratory tract infections mainly in young children.
• HCoV-NL63 shares a common host cell virus receptor (ACE2) with SARS-like coronaviruses.
• HCoV-NL63 is a safe surrogate to study disease mechanisms and develop therapeutic interventions against SARS-like-CoV.

## 1 Introduction

The ongoing COVID-19 pandemic caused by the severe acute respiratory syndrome coronavirus type 2 (SARS-CoV-2) has resulted in ∼6.7 million deaths globally, raising concerns about coronavirus (CoV) infections (WHO, 2021). Although human coronaviruses (HCoVs) were initially thought to cause mild symptoms, such as with HCoV-229E, or HCoV-OC43, new emerging diseases have evolved into severe forms like the original SARS outbreak in 2003 (Drosten et al., 2003; Ksiazek et al., 2003), Middle East respiratory syndrome (MERS) in 2012 (Zaki et al., 2012), and COVID-19 in 2019 (WHO, 2020). Although less severe, other HCoVs have also been recently discovered, including HCoV-NL63 in 2004 (Fouchier et al., 2004; van der Hoek et al., 2004) and HCoV-HKU1 in 2005 (Woo et al., 2005), HCoV-NL63 being the most prevalent HCoV after HCoV-OC43 (Canducci et al., 2008).

Patients affected with HCoV-NL63, primarily develops into mild to moderate respiratory disease symptomatically featuring fever, cough, and runny nose, or into croup and pneumonia (Bastien et al., 2005; Forster et al., 2004; König et al., 2004; Vabret et al., 2005; van der Hoek et al., 2005). HCoV-NL63, like other CoVs, was thought to be a zoonotic pathogen with an origin in bats (Huynh et al., 2012; Tao et al., 2017). Bats not only work as a reservoir but also as a home for interspecies genetic recombination in the development of new genotypes or new emerging viruses (Al-Khannaq et al., 2016; Tao et al., 2017). These genetic changes cause HCoV-NL63 to have a seasonal/repeated infection pattern every year, although with low frequencies of about 2% and self-limiting respiratory infections (Al-Khannaq et al., 2016; Chiu et al., 2005; Kiyuka et al., 2018; Sipulwa et al., 2016).

HCoV-NL63 shares a common host cell virus receptor (ACE2) with SARS-CoV and SARS-CoV-2 (Hofmann et al., 2005). It is also known that both HCoV-NL63 and SARS-like CoVs infect ciliated respiratory cells, although with different efficiency, using the same virus receptor (Dijkman et al., 2013; Banach et al., 2009; Zhu et al., 2020). Working with SARS-like CoVs require access to a high-containment level biosafety laboratory (BSL-3), while HCoV-NL63 work can be performed in BSL-2 facilities. Therefore, HCoV-NL63 can be used as one of the safe surrogates for comparative studies on viral infectivity and replication capacity, disease mechanisms and therapeutic interventions against the emerged SARS-CoV-2. This prompted us to review the current research towards the mechanism of infection and replication of HCoV-NL63.

## 2 Taxonomy

**Fig. 1:**
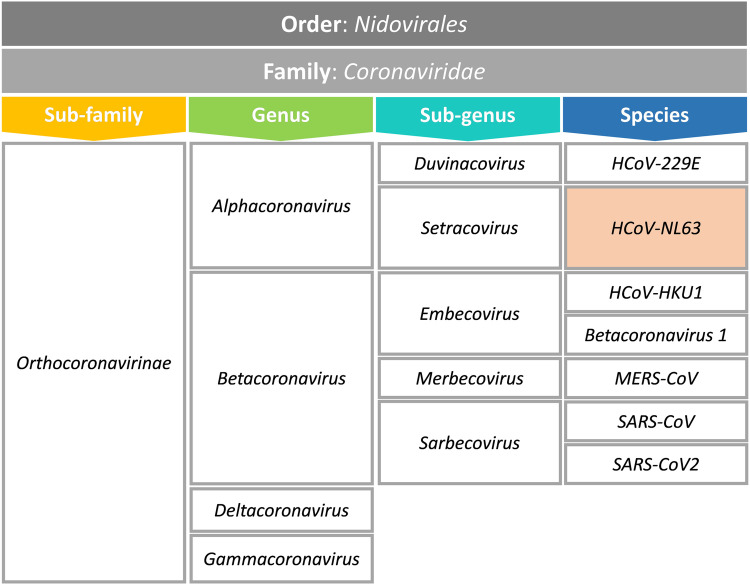
**Taxonomy of HCoV-NL63.** HCoV-NL63 belongs the order *Nidovirales*, the largest single-stranded RNA genomes of positive polarity, family *Coronaviridae*, subfamily *Orthocoronavirinae* which has four genera: *Alphacoronavirus, Betacoronavirus, Deltacoronavirus*, and *Gammacoronavirus*. HCoV-NL63 is included in the subgenus *Setracovirus* within the *Alphacoronavirus* genus.

HCoV-NL63 belongs to the order *Nidovirales*, family *Coronaviridae*, subfamily *Orthocoronavirinae*, genus *Alphacoronavirus*, subgenus *Setracovirus* (ICTV, 2020), a virus order with the largest number of RNA genomes currently known (Fig. 1). HCoV-NL63 is closely related to other alphacoronaviruses, including transmissible gastroenteritis virus (TEGV), porcine epidemic diarrhea virus (PEDV), feline infectious peritonitis virus (FIPV), canine coronavirus (CCoV), and HCoV-229E. The *Orthocoronavirinae* subfamily also includes 3 important zoonotic viruses, all within the genus *Betacoronavirus*: SARS-CoV and MERS-CoV, introduced to humans earlier in the 21st century, and the recently discovered SARS-CoV-2.

**Fig. 2:**
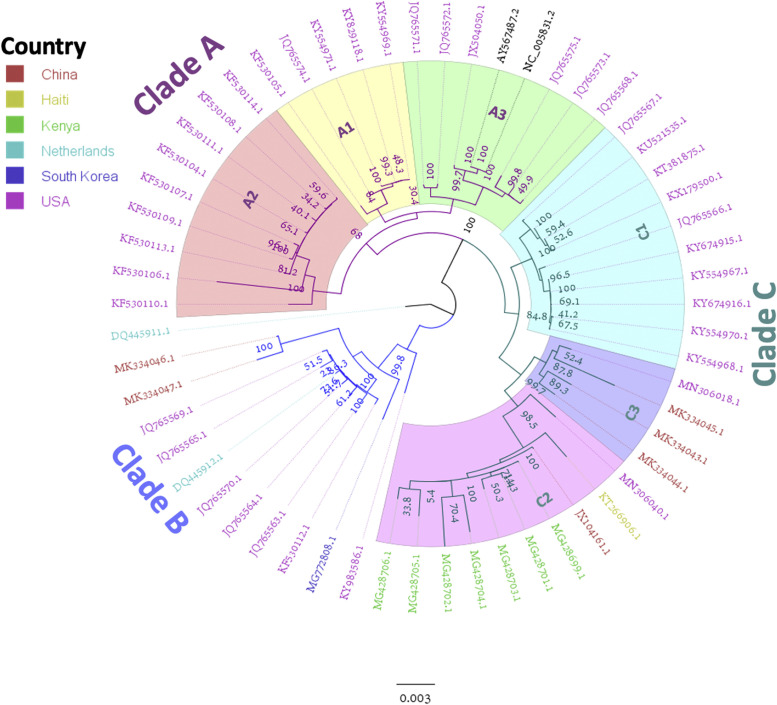
**Phylogenetic analyses based on complete gene sequences of HCoV-NL63.** A total of 58 whole genome sequences of HCoV-NL63 were used for phylogenetic analysis using Geneious Prime® 2021.2.2. Genomes were aligned with MAFFT (v7.450) and Neighbor Joining tree was constructed with PhyML (3.3.20180621) with 1000 Bootstrap. Three clades (A, B and C) were identified with using genome NCBI accession DQ445911.1 as root. Subclades were identified with bootstrap value of over 99% where Clades A and C were clustered into 3 subclades each. FigTree phylogenetic drawing tool (V1.4.4) was used to build the tree.

The recombination between different HCoV-NL63 strains or with other CoVs have resulted in different clades (A, B, C) and seven subclades (A1, A2, A3, B, C1, C2, and C3) described so far. The present review includes an updated phylogenetic analysis on the complete gene sequences of HCoV-NL63 (Fig. 2). The internal variability is mainly based on the amino (N)-terminal domain of the spike gene and the nsp2/nsp3 sequence of the ORF1a (Arden et al., 2005; Chiu et al., 2005; Dominguez et al., 2012; Minosse et al., 2008; Moës et al., 2005; Wang et al., 2020), while the nucleotide sequence from 1b gene is rather conserved (Arden et al., 2005). The different genotypes co-circulate as a mixture of variants strains (Chiu et al., 2005; Minosse et al., 2008), with a prevalence of ∼1–3% among symptomatic patients with respiratory disease, with genotype A associated with most of the hospitalizations due to life-threatening acute respiratory disease (Arden et al., 2005; Dominguez et al., 2012; Minosse et al., 2008). The highly prevalent circulation of HCoV-NL63 might result in widespread, newly emerging subgenotypes showing more severe respiratory symptoms (Wang et al., 2020).

## 3 Virus isolates for research purposes

Different HCoV-NL63 sequences have been submitted to the GenBank database, but few studies reported virus isolation and propagation *in vitro*. Van der Hoek. (2004) was the first isolating the virus (“Amsterdam-1 isolate”) from a nasopharyngeal swab of a 7-month-old child in January 2003 in Amsterdam, Netherlands (van der Hoek et al., 2004). One month later, Fouchier (2004) reported the isolation of HCoV-NL63 from a nasal swab obtained from a 8-month-old child in Rotterdam, Netherlands (Fouchier et al., 2004). Later, Lednicky (2013) isolated the virus from a contaminated stock of renal proximal tubule epithelial cells (RPTEC) in 2004 (Lednicky et al., 2013), while Beau De Rochars (2017) isolated the virus from blood samples of four children of Haiti in 2014–2015 (Beau De Rochars et al., 2017). Most recently, in Japan, Komabayashi (2021) isolated the virus from six frozen nasopharyngeal swabs collected between 2012 and 2020 (Komabayashi et al., 2021). Among all the isolates described in the literature, only the Amsterdam-1 isolate originally described (van der Hoek et al., 2004) is broadly available for the research community via BEI Resources Repository (NIAID, NIH: HCoV-NL63, NR-470), a global provider of materials for infectious disease research, which might limit the pathogenic comparison between studies.

Phylogenetic studies from sequenced viral genomes reported that three genotypes (A, B, C) with probable different disease severity (Dominguez et al., 2012; Shao et al., 2022; Wang et al., 2020). The greatest variability was found in the receptor binding domain (RBD) of the S protein, followed by nsp3. Interestingly, a single mutation within the S protein (I507L) was identified in the subgenotype C3, which was associated with enhancing virus entry in cell culture (Wang et al., 2020).

## 4 Genomic organization

Like other members of the order *Nidovirales*, HCoV-NL63 is an enveloped single-stranded positive-sense RNA virus (27,553 bases in size) that is capped and polyadenylated (van der Hoek et al., 2004). Following the typical Nidovirus genome organization: 5′-ORF1a-ORF1b-spike (*S*)-ORF3-envelope (E)-membrane (M)-nucleocapsid (N)-polyadenylated Tail-3′ (Pyrc et al., 2004), the HCoV-NL63 genome includes a large ORF 1ab (two-thirds of the genome) on the 5′ terminal encoding the proteases and most proteins (non-structural proteins, nsp1-nsp16) necessary for controlling gene expression and replication. The ORF 1a overlaps with the 1b gene region, which encodes an RNA-dependent RNA polymerase (van der Hoek et al., 2006). HCoV-NL63 ORF1a/1b contains a putative elaborated pseudoknot structure (ribosomal frameshifting element between ORF 1a and ORF1b) that triggers a –1 ribosomal frameshift to translate the complete 1ab polyprotein (Namy et al., 2006; Pyrc et al., 2004). Likewise, the 3′ terminal contains the regions for structural proteins (one-third of the genome) S, E, M, and N (van der Hoek et al., 2006). The HCoV-NL63 genome encodes for only one accessory N-glycosylated protein known as ORF3, which is expressed from a distinct subgenomic RNA, one of at least 6 distinct mRNAs (Pyrc et al., 2004). The ORF3 gene has a unique nucleotide composition and appears as a U-rich and A-poor region within the genome, indicating a recent transfer event from another viral or cellular origin (Pyrc et al., 2004).

## 5 Virion structure

Members of family *Coronaviridae* are roughly spherical with virion size ranging from 120 to 160 nm in diameter, and envelop showing petal shaped surface projections of the S protein decorating the surface of the virion (ICTV, 2020). The viral envelope is supported by the M protein and contains a small amount of the E protein (Liu and Inglis, 1991; Schoeman and Fielding, 2019; Yu et al., 1994). Inside the viral envelope, the genome is bound by the nucleocapsid N protein and forms a helical symmetric nucleocapsid (Masters, 2006).

### 5.1 S protein

The HCoV-NL63 S protein mediates both cell attachment and membrane fusion; it is the major determinant of host and tissue tropism and may contribute to viral pathogenesis by activating the endoplasmic reticulum (ER) stress response (Chan et al., 2006; Zheng et al., 2006). The S protein is a large type I single-chain transmembrane glycoprotein, which forms a homotrimer with a molecular weight of 128–160 KDa before glycosylation and 150–200 KDa after N-linked glycosylation (Holmes et al., 1981; Rottier et al., 1981). The HCoV-NL63 S protein is a class I fusion protein similar to influenza virus hemagglutinin and the HIV-1 Env glycoprotein gp120/gp41 (Bosch et al., 2003; Pyrc, 2007; Weis et al., 1988; Weissenhorn et al., 1997). As a class I viral fusion protein, the S protein forms homotrimer and is cleaved by host proteases into the N-terminal subunit (S1; bulb) for receptor binding and the carboxyl-terminal subunit (S2; stalk) for membrane fusion. Disulfide bonds modify the exterior ectodomain of the S protein, whereas the conserved cysteine residues in the very short cytosolic tail are modified by palmitoylation (Fung and Liu, 2018). The variable S1 subunit constitutes the receptor-binding domain (RBD) and contains a unique 179-amino-acid domain not present in other CoVs (Li et al., 2007; van der Hoek et al., 2004). The conserved S2 subunit contains a membrane-spanning region (transmembrane and cytoplasmic domains); 2 generally conserved heptad repeat regions (HR1 and HR2) forming a 6-helix bundle that is critical during virus fusion; and the fusion peptide, which is similar to other class I fusion proteins (van der Hoek et al., 2006).

### 5.2 M protein

The M protein (25–30 KDa) is the most abundant structural protein, embedding in the envelope via 3 transmembrane domains (Masters, 2006). The M protein forms homodimer and interacts with other viral structural proteins to orchestrate the assembly of the CoV particle. In HCoV-NL63 and most other CoVs including the related HCoV-229E, the short N-terminal ectodomain of M protein is modified by N-linked glycosylation (Dea et al., 1990; Hogue and Nayak, 1990; Naskalska et al., 2018; Stern and Sefton, 1982; Utiger et al., 1995). However, in some animal CoVs, like the mouse hepatitis virus (MHV) and the bovine coronavirus (BCoV) or in HCoV-OC43, this ectodomain is modified by O-linked glycosylation (Deregt et al., 1987; Holmes et al., 1981; Lapps et al., 1987; Mounir and Talbot, 1992). The M protein may also contribute to viral pathogenesis. For example, the retinoic acid-inducible gene 1 (RIG-I)-dependent induction of type I interferon (IFN) is observed in cells overexpressing the M protein of SARS-CoV but not HCoV-HKU1 (Siu et al., 2014b).

### 5.3 E protein

The E protein is a small (8–12 KDa) integral membrane protein found in low amounts in the virion (Corse and Machamer, 2000; Liu and Inglis, 1991). Current evidence on avian infectious bronchitis coronavirus (IBV) strongly suggests that the E protein adopts an N-ecto/C-endo topology with one transmembrane domain (Corse and Machamer, 2000; Yuan et al., 2006). In SARS-CoV, the E protein is modified by N-linked glycosylation, and 3 cysteine residues in its endodomain are modified by palmitoylation (Liao et al., 2006; Yuan et al., 2006). Additionally, different studies have shown that the E protein of both SARS-CoV and IBV forms homopentamers with ion channel (IC) activity (viroporins) (Nieto-Torres et al., 2014; Wilson et al., 2004), where the IC activity could modulate the process of virion release and contribute to viral pathogenesis. Although the deletion of the E gene is not lethal for SARS-CoV, the resultant mutant virus was severely defective in virion morphogenesis and attenuated *in vivo,* compared with the wild-type virus (DeDiego et al., 2007).

### 5.4 N protein

The N protein is a multidomain, multifunctional protein essential for viral replication and a number of cellular processes, including RNA packaging, viral genome replication, and evasion of the immune response (Lu et al., 2011; Szelazek et al., 2017; Zhou et al., 2008). The N-terminal domain is responsible for nucleic acid binding, and the C-terminal domain is involved in protein oligomerization. Interestingly, as with SARS-CoV, the HCoV-NL63 N protein is not translocated in the nucleus of infected cells (Zuwala et al., 2015).

The N protein (43–50 KDa) forms a dimer and binds to the genomic RNA in a beads-on-a-string fashion, forming a helically symmetric nucleocapsid (Chang et al., 2006; Fan et al., 2005; Tan et al., 2006). As with other CoVs, the sgRNA coding the HCoV-NL63 N protein is the most abundant among the different viral proteins (Pyrc et al., 2004). In SARS-CoV and other CoVs, the N protein is phosphorylated by cellular kinases such as glycogen synthase kinase 3 (GSK3) and ataxia-telangiectasia mutated and Rad3-related (ATR) kinase (Fang et al., 2013; Wu et al., 2009a). Other modifications such as SUMOylation, ADP-ribosylation, and proteolytic cleavage by caspases have also been demonstrated in the N proteins of some CoVs (Eléouët et al., 2000; Grunewald et al., 2018; Li et al., 2005). Moreover, the N protein of some CoVs (i.e., IBV, SARS-CoV) can affect cell cycle progression, cytoskeleton organization, gene transcription, and apoptosis induction in infected cells (Harrison et al., 2007; Surjit et al., 2006, 2004; Zhao et al., 2006; Zhou et al., 2008). Contrastingly, no significant alteration of cell cycle progression has been reported in HCoV-NL63 (Zuwala et al., 2015).

Multiple crystallization attempts of the full-length HCoV-NL63 N protein have been unsuccessful because the rigid β-sheets within this region restrict its plasticity compared to those of the interrupted β-structures in SARS-CoV and MHV (Szelazek et al., 2017). The significance of the aforementioned differences for nucleic acid binding remains unknown. The HCoV-NL63 nucleoprotein forms oligomers via its C-terminal domain (CTD) and binds nucleic acids via its N-terminal domain (NTD) (Zuwala et al., 2015). The CTD exists as a dimer in solution and tends to aggregate in solution, which may reflect further steps of nucleocapsid assembly (Zuwala et al., 2015). Nevertheless, in the case of the HCoV-NL63 CTD, interaction is weak if present at all (Zuwala et al., 2015). The HCoV-NL63 NTD contains a large, positively charged groove implicated in RNA binding. Each monomer within the structure coordinates several sulfate ions derived from the crystallization buffer. Sulfate ions chemically resemble phosphate moieties present within the structure of RNA and are therefore likely to be located at the same binding sites (Szelazek et al., 2017). HCoV-NL63 NTD has little or no specificity for particular sequences, or even the type of nucleic acid (i.e., RNA vs. DNA) (Zuwala et al., 2015). Interestingly, in the case of the swine alphacoronavirus TGEV, the RNA-binding ability of the N protein is important, not only for genome encapsidation but also for discontinuous transcription and polymerase template switching (Mateos-Gómez et al., 2011; Zúñiga et al., 2010).

### 5.5 ORF3 accessory protein

Poorly characterized overall, the N-glycosylated ORF3 protein has been detected within the ER/Golgi intermediate secretory compartment (ERGIC), where CoV assembly and budding occur. HCoV-NL63 ORF3 is incorporated into virions colocalized with E and M proteins in the same compartment and facing towards the extracellular space. This further suggests an important function, particularly in virus assembly and/or budding from infected cells (Müller et al., 2010).

## 6 Virus entry and replication mechanism

### 6.1 Virus attachment

**Fig. 3:**
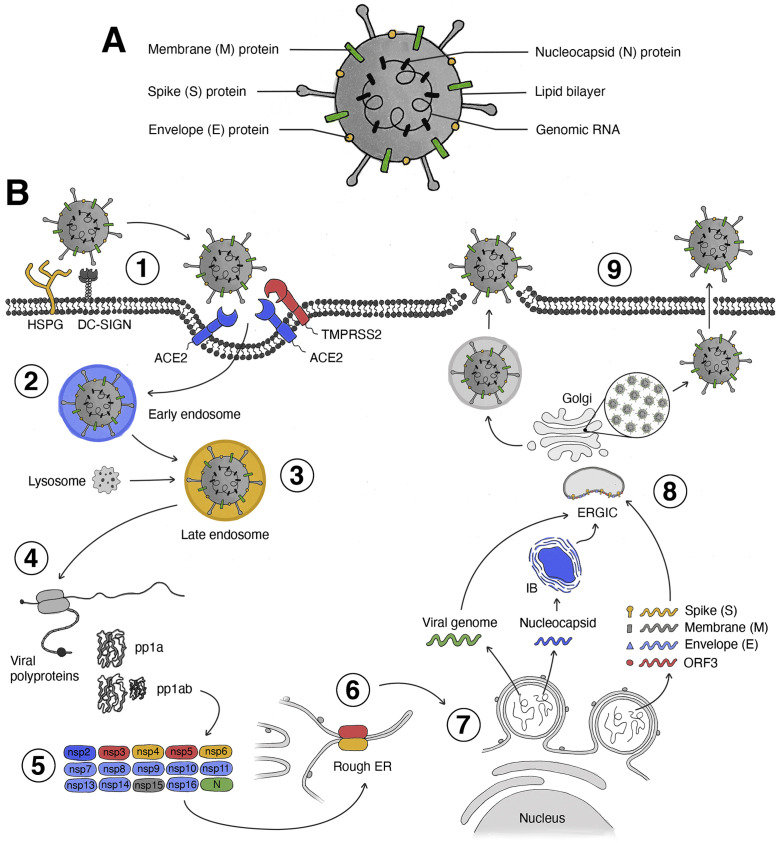
**Life cycle of HCoV-NL63.** a) Virion structure consists of spike (S), membrane (M), envelope (E), nucleocapsid (N), and open reading frame (ORF) 3 protein, with (+) single-stranded RNA genome. b) (1) The virion interacts with the attachment factor heparan sulfate proteoglycans (HSPG) and DC-SIGN to enhance binding of the S protein with the viral receptor ACE2 on surface of target cells. (2) Virus-ACE2 interaction triggers receptor mediated endocytosis, with acidification in the endosome. (3) Spike protein priming is carried out with TMPRSS2 in the early endosome or cathepsins in the late endosome, and viral membranes fuse with the endosome to release the viral genome into the cytoplasm. (4) Nucleocapsid protein is degraded to release the uncoated viral genome ready for translation with the ribosomes to produce the two viral polyproteins (pp) 1a and 1ab through ribosome −1 frameshifting. (5) Viral polyproteins induce autoproteolytically cleavage with their proteases nsp3 and nsp5. (6) Membrane-spanning non-structural proteins (nsp) nsp3 and nsp4 mediates the zippering of the rough endoplasmic reticulum (RER) membrane to generate double membrane structures (DMS). (7) Virus genome and nsps initiates the viral replication and transcription within the DMS, releasing the sgRNA outside to translate the viral structural proteins. (8) N protein accumulated in inclusion bodies (IB), while other structural proteins are processed into the ERGIC. Viral genome release from DMS is coated with N protein and directed to ERGIC and Golgi where new virions are assembled and packaged into vesicles. (9) Virions can be released either via exocytosis or through cell lysis.

The airway epithelium, particularly ciliated and secretory cells of the nasal, bronchial, and alveolar epithelium, constitutes the major target for most known CoVs, including HCoV-NL63 (Dijkman et al., 2013; Sungnak et al., 2020; Ziegler et al., 2020). Respiratory epithelial cells abundantly express angiotensin-converting enzyme 2 (ACE2), the host receptor for HCoV-NL63 and SARS-like CoVs binding and cell entry (Hofmann et al., 2005; Jia et al., 2005; Sims et al., 2005) (Fig. 3). Genome-wide CRIPSR screen in Calu-3 and Caco-2 cells revealed that adaptin AP1G1 and flippase ATP8B1 are general CoV co-factors for HCoV-NL63, HCoV-229E, and SARS-CoV-2. An additional co-factors more specific for HCoV-NL63 was the histone acetyltransferase EP300 regulating ACE2 expression (Rebendenne et al., 2021).

The HCoV-NL63 S protein shares more than 50% of amino acid (aa) identity with the HCoV-229E, and both viruses belong to the same genus, but they use different viral receptors (Pöhlmann et al., 2006). Conversely, HCoV-NL63 S protein shares only 25% and 17.1% of aa sequence identity with SARS-CoV and SARS-CoV-2, respectively. However, these 3 viruses use ACE2 as a host receptor with different binding affinities (Brielle et al., 2020; Smith et al., 2006). The RBD of the HCoV-NL63 S-protein has 3 non-linear binding motifs located at the C-terminus S1 subunit (aa 476–616), and these in conjunction with distinct aa from the central regions of the CTD of S1 interact with ACE2 (Hofmann et al., 2006; Lin et al., 2008; Pöhlmann et al., 2006). These regions largely overlap based on sequence homology and show comparable binding affinities with SARS-CoV RBDs (Li et al., 2007; Lin et al., 2008), probably because of the linear and monomeric nature of both RBDs, indicating that in the full-length S-protein of HCoV-NL63, the non-linear RBD has weaker interaction than linear RBD from SARS-CoV (Mathewson et al., 2008; Wong et al., 2004; Wu et al., 2011). Initial proteolytic studies of the S2 fusion core identified an α-helical domain consisting of a trimer of the HR segments N57 and C42. The resolved crystal structure of this trimer complex shows distinctive high-affinity conformations of interacting cross-sectional layers of 6 helices. It has been suggested that the larger HR regions of the alphacoronavirus may be required to prime the S proteins for the fusion-activating conformational changes during entry of the virus (Zheng et al., 2006).

Variations in aa composition and glycosylation are crucial for CoV infectivity and interaction with the host immune system. In HCoV-NL63, a deletion of 18 aa from the C-terminus of the S protein, corresponding to an intracellular retention signal, has been shown to enhance (1.5 times) its accumulation and facilitating virus entry. Further deletion of residue 29 has also been shown to enhance the amount of S protein on both the cell surface and virion yet reducing virus entry by 25%, suggesting that residues 19–29 may contribute to membrane fusion. A 29 aa-deletion mutant had a defect in anchoring on the plasma membrane, which led to a dramatic decrease of S protein in virion and virus entry; a total of 15 residues (Y498, V499, V531, G534, G537, D538, S540, G575, S576, E582, W585, Y590, T591, V593, and G594) within the RBD were necessary for receptor binding and virus entry (Lin et al., 2011). They probably form 3 receptor-binding motifs, being the 3rd motif highly conserved between HCoV-NL63 and SARS-CoV (Lin et al., 2011; Wu et al., 2009b). Ren et al. (2022) reported that ACE2 polymorphisms could impact susceptibility to infection by SARS-CoV-2, SARS-CoV, and HCoV-NL63. For example, ACE2 variants D355N and G352V restrict the S protein-ACE2 interaction and limit infection, where HCoV-NL63 S protein binds to ACE2 less efficiently (Ren et al., 2022).

Furthermore, the C-terminal region (aa 153–226) of the HCoV-NL63 M protein is responsible for attaching heparan sulfate proteoglycans to target cells (Milewska et al., 2014; Naskalska et al., 2019), and C-type lectin receptors like DC-SIGN could work as co-receptors (Hofmann et al., 2006). Most recently, genome-wide CRISPR screens in Vero-E6 identified HMGB1 as a novel regulator of ACE2 involved in virus entry of HCoV-NL63, SARS-CoV, and SARS-CoV-2. This nuclear protein translocates to the cytoplasm under cellular stress conditions (Wei et al., 2021).

### 6.2 Endocytosis

After HCoV-NL63 attachment to host cell receptors, virions undergo clathrin-mediated endocytosis, which is pH-sensitive and requires acidification of the endosome, subsequent severance by dynamin, leading to viral genome release (Hofmann et al., 2005; Huang et al., 2006; Milewska et al., 2018b) (Fig. 3), similar to what has been described for HCoV-229E, and SARS-CoV (Blau and Holmes, 2001; Hofmann et al., 2004; Simmons et al., 2004). Whether HCoV-NL63 enters cells through early endosomes using cell surface proteases (e.g., TMPRSS2, TMRSS11D, ADAM17) or late endosomes using lysosomal proteases (cathepsins) remains unclear. Different studies have suggested that clinical isolates of HCoV-229E, HCoV-OC43, and HCoV-HKU1 prefer cell surface proteases to cathepsins. Conversely, lab-adapted viruses use cathepsins for cell entry (Bertram et al., 2013, 2011; Shirato et al., 2017, 2018), which could explain the low infectivity of lab-adapted viruses in human airway epithelium (HAE) cell cultures. It is noteworthy that proteases like proprotein convertase (e.g., furin, PACE4, PC4, PC5, PC7), which activates MERS-CoV, and SARS-CoV-2 but not SARS-CoV (Hoffmann et al., 2020a; Millet and Whittaker, 2014; Shang et al., 2020), are less likely to exert an influence on HCoV-NL63 entry, given that the S protein lacks a furin motif (arginine-rich residues) to be activated (Pyrc et al., 2007). The role of other non-membrane-associated proteases like elastase or coagulation factor Xa, which enhance SARS-CoV infectivity through cleavage of the S protein (Du et al., 2007; Matsuyama et al., 2005), has not been investigated in HCoV-NL63 infections.

Generally, once the virions are internalized into endosomes, the interaction between viral N protein and cellular host proteins is required to release the viral genome from its capsid, which has not yet been demonstrated for HCoV-NL63 (Fig. 3). It has been proposed for HCoV-229E and IBV that valosin-containing protein (p97) would help in the maturation of early endosomes and decomposition of the nucleocapsid (Wong et al., 2015). The mechanism of the subsequent transduction response after ACE2 engagement in relation to HCoV-NL63 pathogenesis has not been investigated.

### 6.3 Genome translation

Like other CoVs, the HCoV-NL63 non-segmented positive RNA genome cap binds ribosomes to initiate the translation of ORF1a and produce polyprotein pp1a (replicase 1a), which contains nsp1 to nsp11 (Fig. 3). At the end of the nsp10 in ORF1a, it is localized in an RNA pseudoknot that enables ribosomes to undergo a –1 frameshifting to generate replicase 1ab, which contains nsp1 to nsp16 (Pyrc et al., 2005). The exact frameshifting efficiency remains unknown for many CoVs, including HCoV-NL63. However, some CoVs like MHV have shown 48–69% efficiency (Irigoyen et al., 2016) or 45–70% efficiency for SARS-CoV-2 (Finkel et al., 2021).

Following translation, the HCoV-NL63 polyprotein contains two nsp with proteolytic domains: nsp3 and nsp5 (Pyrc et al., 2005). These proteases autocatalytically cleavage the polyproteins into 16 nsp (Pyrc et al., 2004). The nsp3 of HCoV-NL63, HCoV-229E, HCoV-OC43, and HCoV-HKU1 contains two proteolytic domains, known as papain-like protease 1 (PLP1pro), which autocleavage the polyprotein at the nsp1/nsp2 site to release nsp1, and PLP2pro, that cleaves polyproteins at the nsp2/nsp3 and nsp3/nsp4 sites to release nsp2 and nsp3, respectively (Chen et al., 2007). Moreover, although less efficient than PLP1, PLP2 can also autocleavage the polyprotein at the nsp1/nsp2 site (Lim and Liu, 1998; Ziebuhr et al., 2007). Other CoVs like MERS-CoV or SARS-CoV contain only one proteolytic domain (PLpro) within the nsp3 (Ziebuhr et al., 2000). The second protease nsp5, known as the main protease (Mpro) or 3-Chymotrypsin-like protease (3CLpro), is a highly conserved endopeptidase with a serine‑like domain responsible for cleaving and releasing the remaining 13 non-structural proteins (Piñón et al., 1999; Pyrc et al., 2005; Ziebuhr et al., 2000). The HCoV-NL63 Mpro is active when forming homodimers containing 3 domains each, where the catalytic site (Cys144 and His 41) is located in a cleft formed between Domain I and Domain II, while Domain III allows the homodimer formation (Wang et al., 2016). Further analysis of substrate specificities of Mpro of HCoV-NL63 found histidine at position P1 instead of glutamine present in other CoVs (Wang et al., 2016). Overall, CoV 3C-like protease substrate profiling has identified glutamine preference for the P1 position, leucine at the P2 position, basic residues at the P3 position, small hydrophobic residues at the P4 position, and small residues at the P1′ and P2′ positions (Chuck et al., 2011).

A genome-wide CRISPR knockout screen in Huh-7.5 found TMEM41B as a pan-CoV host factor required for a post-entry step in the CoV life cycle. This poorly studied ER transmembrane protein was the only gene implicated in autophagy. The specific host factors of HCoV-NL63 rely on a core set of host chromatin regulators like EP300, KMD6A, KMT2D, MED23, MED24, MEN1, PAXIP1, and SETDB1, which were proposed to reprogram the host transcriptome for successful infection (Schneider et al., 2021).

### 6.4 Replication compartments

Similar to other CoVs like IBV, MHC, MERS-CoV and SARS-CoV, HCoV-NL63 generates an ER-derived network such as double-membrane vesicles (DMVs), convoluted membranes (CM), and double-membrane spherules (DMSs) (Knoops et al., 2008; Orenstein et al., 2008; Oudshoorn et al., 2017; Snijder et al., 2020) (Fig. 3). Within these double-membrane structures is where replication and transcription occur to avoid innate immune recognition (Knoops et al., 2008; Snijder et al., 2020). Three membrane-associated non-structural proteins, i.e., nsp3, nsp4, and nsp6, that together induce the formation of the double-membrane compartments (Hagemeijer et al., 2014). The non-structural proteins nsp3 and nsp4 recruit nsp6 to start the process of anchoring more nsps, thus creating the replication-transcription complex (RTC) within the double-membrane compartments (Kanjanahaluethai et al., 2007; Oostra et al., 2008). Host molecules like Golgi-specific brefeldin A-resistance guanine nucleotide exchange factor 1 (GBF1) and ADP ribosylation factor 1 (ARF1) are also required for the double-membrane formation for MHV virion (Verheije et al., 2008).

Nsp3 is a multifunctional protein involved in viral replication (Lei et al., 2018; Neuman et al., 2008). Among its eight domains, the ubiquitin-like domain (Ubl1) facilitates RNA synthesis through binding nsp3 and RTC to the viral genome (Serrano et al., 2007) via a serine‑ and arginine-rich linker region of the residual N protein (Hurst et al., 2013; Keane and Giedroc, 2013), and promotes the initiation complex (Verheije et al., 2010; Zúñiga et al., 2007).

### 6.5 Replication and transcription

As in IBV, MERS-CoV, and SARS-CoV infection, HCoV-NL63 RNA synthesis occurs within replication compartments (Snijder et al., 2020), and the replication is mediated by the common negative-strand intermediates to make either viral genome for new virions and sgRNAs for coding structural proteins through a continuous or discontinuous strategy, respectively (Pyrc et al., 2004) (Fig. 3). Both processes require the formation of the RTC assembled by the recruitment of many nsps and then binding to the 3′-end of the viral genome within the replication compartments (Züst et al., 2008). The precise mechanism of how the RTC switches to generate a viral genome or sgRNAs in HCoV-NL63 replication has not yet been elucidated. However, a proposed model in TGEV has suggested that N protein facilitates sgRNAs transcription (Zúñiga et al., 2010) and that the viral genome synthesis in MHV requires phosphorylation of the viral nucleocapsid by the host GSK-3 with the help of a helicase (Wu et al., 2014).

The generation of sgRNAs in alphacoronavirus is initiated with the formation of short RNA primers (∼6 nucleotides) by nsp7 and nsp8, which are essential co-factors for the viral RNA polymerase (nsp12) (Xiao et al., 2012). To maintain fidelity on CoV genomes like SARS-CoV, the exoribonuclease nsp14 functions as a proofreading enzyme (Minskaia et al., 2006), highly enhanced by nsp10 (Bouvet et al., 2012; Ma et al., 2015). Together, these proteins are part of the RTC, which recognizes specific sequences on the viral RNA to conduct replication or transcription. The RTC recognizes the anti-leader sequence at the 3′-end and starts synthesizing the negative-strand intermediates but pauses at transcription regulatory sequences (TRSs) on the genome (leader-TRS) for making templates for new genomes, or between them (body-TRS), to generate templates for sgRNAs (Pyrc et al., 2007). These negative-strand intermediates are synthesized at very low amounts, at around only 1% of the positive strands like in TGEV (Sethna et al., 1991), and they contain both polyuridylate and anti-leader sequences. Additionally, nsp9 might stabilize newly forming RNA strands to avoid their degradation (Egloff et al., 2004; Sutton et al., 2004).

Although negative strands produce some double-strand intermediates, nsp13 has been found to have C-terminal superfamily-1 helicase domains in HCoV-229E to continue transcription (Seybert et al., 2000). Once positive-strands are formed in SARS-CoV, they undergo 5′ capping through RNA phosphorylation with nsp13 (Ivanov and Ziebuhr, 2004) and follow methylation with nsp14 and nsp16 in association with nsp10 (Bouvet et al., 2010, 2012; Chen et al., 2011; Decroly et al., 2011). Polyadenylation is also carried out at the 3′ end through the adenylyl transferase activity of nsp8 as found in HCoV-229E (Tvarogová et al., 2019).

As for MHV and SARS-CoV-2, once newly formed HCoV-NL63 RNA strands are synthesized, nsp3 mediates the formation of a molecular pore in -infected cells (Wolff et al., 2020). Viral pores allow controlled release of viral RNA transcripts from replication compartments to the cytosol for protein translation. Finally, the translation of the viral proteins is initiated, and the HCoV-NL63 S protein is incorporated into virions through microtubule interaction near the nucleus (Rüdiger et al., 2016), while the RNA-binding N protein exhibits a tendency to aggregate into the cytoplasm, suggesting virion assembly (Zuwala et al., 2015) (Fig. 3).

#### 6.5.1 Cell susceptibility to infection *in vitro*

Several cell lines derived from the kidneys, lungs, or intestines, including LLC-MK2, Vero E6, Vero B4, Vero FM, MRC-5, Caco-2, tertiary monkey kidney cells, renal proximal tubule epithelial cells, human renal epithelial cells, and human airway epithelial cells (HAE), are permissive of HCoV-NL63 growth (Fouchier et al., 2004; Herzog et al., 2008; Komabayashi et al., 2021; Lednicky et al., 2013; Schildgen et al., 2006; van der Hoek et al., 2004) (Table 1). While the expression of ACE2 receptors in these cells correlates with the permissiveness of HCoV-NL63 infection (Milewska et al., 2014; Smith et al., 2006), the soluble form of ACE2 might abrogate infection (Hofmann et al., 2005; Jia et al., 2009), constituting a potential non-functional truncated form of ACE2 (Onabajo et al., 2020).

**Table 1:** Susceptible cells to HCoV-NL63.

Cell type	Description	Purpose	Method of evaluation	Refs.
tMK	Monkey Kidney epithelial, tertiary Monkey kidney cells, *Macaca fascicularis*	Isolation	CPE^a^, EM^b^	Fouchier, 2004; van der Hoek, 2004
Vero	Monkey Kidney epithelial, *Cercopithecus aethiops*	Propagation	CPE, EM, RT-qPCR^c^	Fouchier, 2004
LLC-MK2	Monkey Kidney epithelial, *Macaca mulatta*	Propagation	CPE, EM, IFA^d^, RT-qPCR	van der Hoek, 2004; Hofmann, 2005; Schildgen, 2006; Banach, 2009
Vero E6	Monkey Kidney epithelial, *Cercopithecus aethiops*	Propagation	CPE, RT-qPCR	Lednicky, 2013; Herzog, 2008
Vero B4	Monkey Kidney epithelial, *Cercopithecus sabaeus*	Propagation	CPE, RT-qPCR	Schildgen, 2006
Vero FM	Monkey Kidney epithelial	Propagation	CPE, RT-qPCR	Herzog, 2008
Caco-2	Human Colon epithelial, *Homo sapiens*	Propagation	CPE, RT-qPCR	Herzog, 2008
Huh-7	Human Liver epithelial, *Homo sapiens*	Propagation	CPE, RT-qPCR	Hofmann, 2005
WI-38	Human Lung fibroblast, *Homo sapiens*	Propagation	CPE	Lednicky, 2013
RPTEC	Human Kidney epithelial, *Homo sapiens*	Propagation	CPE, EM, RT-qPCR	Lednicky, 2013
HRE	Human Kidney epithelial, *Homo sapiens*	Propagation	CPE, RT-qPCR	Lednicky, 2013
NHBE	Human Lung epithelial, Normal Human Bronchial Epithelial cells, *Homo sapiens*	Isolation/ Propagation	CPE, EM, IFA, RT-qPCR	Dijkman, 2013; Banach, 2009

a Cytopathic effect (CPE) is characterized with rounded cells and cytoplasmic stranding, clump and detachment of dead cells. The first signs of CPE mostly appeared between 48 - 72 hpi with variations according to the titer of the viral inoculum.^b^ Electron microscopy (EM).^c^ Reverse Transcriptase quantitative PCR.^d^ Immunofluorescence assay.

HCoV-NL63 is hardly more isolated than other CoVs with variable replication kinetics (Dijkman et al., 2013), and mild cytopathic effect (CPE) if present, which can be visualized throughout the cell culture, with a refractive, rounding appearance and vacuolization, followed by detachment (Fouchier et al., 2004; Herzog et al., 2008; Lednicky et al., 2013; Milewska et al., 2014; Orenstein et al., 2008; Schildgen et al., 2006; Smith et al., 2006; van der Hoek et al., 2004). In these studies, the first signs of CPE start between 24–72 h post-inoculation (hpi) and peak between 72 and 96 hpi, with around 25 - 50% of infected cells and an estimated yield of ∼10^5^ TCID_50_/mL. Trypsin treatment showed no enhancement of the infection, and infected freeze-thawed cells were thought to have less infectivity than supernatant alone (Lednicky et al., 2013).

Differentiated human bronchial epithelial cell cultures of HCoV-NL63 revealed a polarized apical release of new virions (Dijkman et al., 2013), with viral proteins nsp3 and nsp4 localized in discrete foci of ciliated cells showing punctuated and perinuclear distribution (Chen et al., 2007; Banach et al., 2009), but also found at the base of the cilia. Immature virions are packaged in vesicles near the rough ER (RER), associated with double-membrane vesicles or areas of granular nucleocapsid material (Lednicky et al., 2013). Assembly seems to occur through sequential budding of virions from a nucleocapsid lined membrane, a pinching off, and then envelope acquisition, with further accumulation of mature virion-laden vesicles into RER or Golgi cisternae that might rupture and release virions into the cytosol, resulting in cell death through either necrosis or apoptosis (Orenstein et al., 2008; Banach et al., 2009).

#### 6.5.2 Infection models *in vivo*

Precedents of *in vivo* infection models for HCoV-NL63 are scarce. In a previous study, Bently et. al. (2021) reported the use of K18-hACE2 mice. The virus was initially propagated *in vitro* in Caco-2 cells and inoculated intranasally in 8–10 weeks-old mice. The inoculation triggered a classical acute inflammatory response in lungs, and active viral propagation that reached plateau levels at 4 days post-inoculation. Moreover, a more recent study reported the use of immunocompromised (STAT1-/-) or sensitized (IFNAR-/-) mice transduced with the viral receptor hACE2 to generate an infection model for HCoV-NL63 (Liu et al., 2023). This study described that BALB/c mice were more susceptible to HCoV-NL63 infection than C57BL/6 mice, and both models were capable of produce specific CD8+ T cells against the virus N protein.

## 7 Virus-host interactions: induction and evasion of host innate immunity

The structural analysis of HCoV-NL63 S protein revealed that the RBD is buried or masked by glycosylation in a native trimer conformation, which might protect the virus from host immune recognition (Walls et al., 2016). However, when recognized by antigen-presenting cells, HCoV-NL63 can trigger a strong cytokine and chemokine mediated immune response, which self-limits the spread of the infection (Lister, 2009).

The S protein of HCoV-OC43, HCoV-HKU1, or SARS-CoV triggers ER stress, and activates unfolded protein response (UPR) in virus-infected cells (Favreau et al., 2009; Siu et al., 2014a). This signaling pathway eventually leads to apoptosis to limit viral replication (DeDiego et al., 2011; Favreau et al., 2009), although not yet studied in HCoV-NL63 (Chan et al., 2006). Apoptosis is also mediated by MAP kinases during CoV infection with HCoV-229E, MERS-CoV, and SARS-CoV (Kono et al., 2008; Mizutani et al., 2005; Yeung et al., 2016).

The role of autophagy in CoV replication is not clear since virus can manipulate cell machinery for DMV formations (Reggiori et al., 2010). Infected cells with TGEV activates autophagy cells to protect from oxidative stress and avoid apoptosis (Zhu et al., 2016), but promotes viral replication in certain cases like in PEDV infection (Guo et al., 2017). The activation of autophagy depends on the activation of the microtubule-associated protein 1A/1B-light chain 3 (LC3) by the HCoV-NL63 PLP2 transmembrane protein (TM), independently of the catalytic site (Chen et al., 2014). Although the catalytic site has a robust deubiquitinating action on STING, RIG-I, TBK1, and IRF-3 to avoid IFN signaling activation, it is not required to antagonize the STING-mediated activation of IRF-3 (Chen et al., 2007; Clementz et al., 2010; Nicholson et al., 2008; Sun et al., 2012). PLP2-TM interacts with Beclin1 to inhibit STING and impair the autophagosomes' maturation through interference with lysosome fusion (Chen et al., 2014). HCoV-NL63 PLP2-TM also promotes IFN inhibition through ubiquitination of the cellular oncoprotein MDM2. The stabilized form of MDM2 induces proteasomal degradation of p53, which is responsible for activating IRF7 (Yuan et al., 2015).

Host protein shutdown mediated by nsp1 is another strategy commonly used by different HCoVs, including MERS-CoV, SARS-CoV, HCoV-229E, and HCoV-NL63 during replication and propagation in their host cells (Jauregui et al., 2013; Krähling et al., 2009; Lokugamage et al., 2015; Narayanan et al., 2008). HCoV-NL63 (or HCoV-229E) nsp1 interacts with the host ribosomal 40S subunit, blocking mRNA binding, inhibiting cell protein synthesis (Wang et al., 2010). Despite the fact that IRF-3 phosphorylation is not affected, interferon signaling is inhibited transcriptionally but mainly translationally. Furthermore, nsp15 (EndoU), although it has not yet been studied in HCoV-NL63, is conserved among CoVs (e.g., MHV and PEDV) and shows an endoribonuclease activity that excises the 5′ poly-uridine sequence from negative RNA strands, avoiding host MDA5 recognition and associated interferon response (Hackbart et al., 2020). The innate immune evasion also occurs through Mac1 domain within the nsp3 of MHV, HCoV-229E, or SARS-CoV countering IFN mediated responses such as ADP-ribosylation, and facilitating viral replication (Fehr et al., 2015; Grunewald et al., 2019; Li et al., 2016).

## 8 Antiviral strategies

Effective antiviral treatment is required when HCoV-NL63-infected patients undergo clinical complications. Despite the fact that intravenous administration of immunoglobulins seemed to be protective (Pyrc et al., 2006), they might not be available for high numbers of patients during outbreaks. Thus, different strategies have been proposed to suppress HCoV-NL63 replication by either targeting the virus or enhancing host antiviral mechanisms (Table 2).

**Table 2:** Antiviral agents against HCoV-NL63.

Antiviral	Target	Mechanism	Refs.
Oligopeptides derived from RBD	ACE2	Competitive binding	Struck et al., 2012
Chitosan derivatives, Thymoquinone	ACE2	Receptor blocking	Milewska et al., 2013, 2016; Xu et al., 2021
Brilacidin	HSPGs	Entry inhibition	Hu et al., 2022
Anti-sense oligonucleotides (ASO)	ACE2	Receptor downregulation or modulation	Rehman et al., 2020
Iminosugars	ER glycan processing	ER glucosidase inhibition	Zhao et al., 2015
Oligopeptides derived from HR2	HR1	Competitive binding	Pyrc et al., 2006
Camostat	TMPRSS2	Protease inhibition	Kawase et al., 2012
Tryptanthrin	PLP2pro	Protease inhibition	Tsai et al., 2020
Caffeic acid	ACE2	Competitive binding	Weng et al., 2019
Michael aceptor inhibitor N3	Nsp5	Protease inhibition	Yang et al., 2005
Pyrimidine nucleoside analogue	Nsp12	Replication inhibition	Pyrc et al., 2006
Fleximer 2	Nsp12	Replication inhibition	Peters et al., 2015
IFITM proteins	Endosome	Virus entry inhibition	Wrensch et al., 2014
Immunophilins inhibitors	Calcineurin	Cellular immunosupression	Pfefferle et al., 2011; Carbajo-Lozoya et al., 2014, 2012
APOBEC3	Cytidine	Genome editing	Milewska et al., 2018
Boceprevir, Calpain inhibitor II, XII, and GC-376, Dyphylline, Naphthoquine, AT7519, calpeptin, ifenprodil, MUT056399, pelitinib, tolperisone, and triglycidyl isocyanurate, Shikonin	Nsp5	Protease inhibition	Hu et al., 2021; Song et al., 2022; Wang et al., 2022b; Günther et al., 2021; Zhang et al., 2022
Stenoparib	Poly (ADP-ribose) polymerase	Virus entry inhibition	Stone et al., 2021

### 8.1 Receptor blockers

The similarities between RBD of HCoV-NL63 and SARS-CoV have revealed a cross-inhibition of the ACE2 transduction by the S protein (Wu et al., 2011). For instance, in one study, a linear peptide (YKYRYL) derived from the SARS-CoV RBD was able to block the interaction of HCoV-NL63 S protein with the ACE2 receptor, being the KYR motif critical for binding (Struck et al., 2012). Other studies have demonstrated that the interaction of HCoV-NL63 S protein and ACE2 could be prevented by using N-(2-hydroxypropyl)−3-trimethylammonium chitosan chloride (HTCC), a chitosan derivative with antisense oligonucleotides targeting residues of ACE2 responsible for its interaction with RBD (Milewska et al., 2013, 2016) or with phenolic compounds like caffeic acid from plant extract (Weng et al., 2019). The phytochemical compound Thymoquinone from *Nigella sativa* binds to ACE2 to block the entry of HCoV-NL63, SARS-CoV, and SARS-CoV-2 (Xu et al., 2021). In another example, methylene blue inhibits the interaction between SARS-CoV-2 and HCoV-NL63 spike protein and ACE2, IL-2R, TNFR, or CD40, including delta (B.1.617.2) variant through entry and replication inhibition (Chuang et al., 2022).

The use of splice-switching antisense oligonucleotides modulates alternative isoforms of ACE2 that limit infection (Rehman and Tabish, 2020). Alternately, the inhibition of ER glucosidases with iminosugars impairs the N-glycosylation and transduction of ACE2, but it also affects ACE2 expression and virion production (Zhao et al., 2015).

The heptad repeats (HR1 and HR2) of the S protein are critical during the fusion step of virus entry in conjunction with host proteases like TMPRSS2 for S protein priming. Therefore, they make attractive targets for the development and assessment of entry inhibitors. Heptad repeats' (HRs') interaction with the spike protein is also required to promote viral membrane fusion. Heptad repeat-derived peptides from HR2 are designed to block HR interaction (Pyrc et al., 2006), while the selective cysteine protease inhibitor-camostats antagonize TMPRSS2 (Hoffmann et al., 2020b; Kawase et al., 2012).

Host defense peptides (HDPs) are typically 12- 50 aa length expressed in neutrophils and mucosa, and serve as the first line of defense against foreign pathogens. Brilacidin, a synthetic HDP, demonstrates broad-spectrum antiviral against HCoV-OC43, −229E, -NL63, and SARS-CoV-2 but not influenza or enterovirus (Hu et al., 2022) through blocking virus attachment and early entry targeting heparan sulfate proteoglycans (HSPGs).

### 8.2 Replication blockers

Viral proteases develop a critical role in polyprotein processing, which is essential for viral replication. The HCoV-NL63 PLP2 shares substrates with other CoVs like SARS-CoV, but understanding the differences therein could drive the development of potent and selective inhibitors (Báez-Santos et al., 2014). For example, the inhibitor GRL0617 binds the catalytic site of SARS-CoV PLpro to produce inversion in the six-residue loop (G267-NYQC-G272) between residues Tyr269 and Gln270, but this does not occur on HCoV-NL63 PLP2 where different residues (G253-SFDN-G258) are used (Chaudhuri et al., 2011). A large screen of antiviral compounds from main protease from SARS-CoV-2 identified 37 compounds that inhibit PLpro, with seven (i.e., AT7519, calpeptin, ifenprodil, MUT056399, pelitinib, tolperisone, and triglycidyl isocyanurate) exhibiting >100-fold virion reduction and very low cytotoxicity (Günther et al., 2021).

Furthermore, natural active molecules like tryptanthrin or shikonin have been proposed as HCoV-NL63 PLP2 and SARS-CoV-2 Mpro inhibitors (Tsai et al., 2020; Zhang et al., 2022), and also inhibition on binding of SARS-CoV-2 S1 to ACE2 (Hagiyama et al., 2022). Large screening of natural products against pan-CoV main protease identified 12 compounds *in vitro* such as hypericin, rosmarinic acid, isorhamnetin, and luteolin for SARS-CoV-2 main protease (Shahhamzehei et al., 2022).

Other inhibitors like Michael acceptor N3 target the main protease (nsp5), which is highly conserved among CoVs (Wang et al., 2016; Yang et al., 2005). For the active replication, some nucleoside analogs have been developed for HCoV-NL63, such as the pyrimidine nucleoside analogs β-D-N4-hydroxycytidine and 6-azauridine (Pyrc et al., 2006) and Fleximer 2, which have shown good inhibition (Peters et al., 2015).

Boceprevir, Calpain inhibitor II and XII, GC-376, dyphylline, and naphthoquine have shown broad-spectrum antiviral activity against CoV main protease, inhibiting both viral Mpro and host cathepsin L, with additive antiviral effect when combined with remdesivir (RNA-dependent RNA polymerase inhibitor) (Hu et al., 2021; Song et al., 2022; Wang et al., 2022b). Although, recently identified protease inhibitor, GC376 or Nirmatrelvir, shows good promising results than boceprevir in inhibiting SARS-CoV-2 and HCoV-NL63, −229E, and -OC43 replication (Wang et al., 2022a; Weil et al., 2022).

With regard to replication blockers, Stenoparib is an inhibitor of cellular poly (ADP-ribose) polymerase (PARP) that blocks SARS-CoV-2 and HCoV-NL63 replication by reducing virus entry and complete inhibition of plaque formation. In addition, this drug showed a synergistic effect with remdesivir with more than 90% virus suppression on HCoV-NL63 (Stone et al., 2021).

### 8.3 Other antiviral strategies

Among the host factors, the interferon-inducible transmembrane (IFITM) proteins inhibit the entry of most CoVs, including HCoV-NL63, HCoV-229E, and SARS-CoV, probably through cholesterol accumulation on late endosomes (Huang et al., 2011; Wrensch et al., 2014). However, it has the opposite effect in other CoVs like HCoV-OC43 (Zhao et al., 2014). These differences are explained by differential response of S proteins with specific motifs in IFITMs that control viral entry (Zhao et al., 2018).

When a virus reaches the cytoplasm, the ubiquitous immunophilins play an important role in controlling HCoV-NL63 replication. Immunosuppressive ligands like cyclosporine A and FK506 (tacrolimus) or non-immunosuppressive derivatives like alisporivir and NIM811 have shown inhibitory effects for HCoV-NL63, HCoV-229E, and SARS-CoV (Carbajo-Lozoya et al., 2014, 2012; de Wilde et al., 2011; Pfefferle et al., 2011). Another important enzymatic superfamily is APOBEC3, from which the A3C, A3F, and A3H members have shown viral inhibition (Milewska et al., 2018a), but the exact inhibitory mechanism has still not been discovered.

The alkalizing molecule and autophagy inhibitor ROC-325 blocks lysosome acidification. The partial siRNA knockdown of ATP6V0D1 reduced LLC-MK2 cells HCoV-NL63 CPE by 60% highlighting the role of blocking acidification to inhibit co-infection (Gorshkov et al., 2021).

Novel anti-SARS-CoV-2 fusion inhibitory IPB19 lipopeptide derivatives based on MPER (membrane-proximal external region) peptide from the S protein also cross inhibited other HCoVs including HCoV-NL63 (Yu et al., 2021).

### 8.4 Cross-protection between HCoV-NL63 and other coronaviruses

Overall, literature review shows that baseline antibodies to common human coronaviruses like OC43, HKU1, 229E, and NL63 are not associated with cross-neutralization and potential protection against SARS-CoV-2 infection. Authors found a correlation between the antibody levels of SARS-CoV-2 N protein and the severity of the disease, but not correlation with antibody levels to other low pathogenic seasonal coronaviruses (HCoV-OC43, HCoV-HKU1, HCoV-229E, and HCoV-NL63) (Adams et al., 2022). Using neutralization assays, antibodies generated against HCoV-OC43, HCoV-NL63, and HCoV-229E seemed not to be protective against SARS-CoV-2 infections in young children (Dhochak et al., 2022). However, Wells et al. (2022) reported that SARS-CoV-2 infection or vaccination boosted the level of NL63 neutralizing antibodies. Moreover, it was reported that SARS-CoV-2 and HCoV-NL63 neutralizing antibodies waned over time, although vaccination support protection against SARS-CoV-2 but not to HCoV-NL63 (Hastert et al., 2022). Vaccination against SARS-CoV-2 showed efficient cross-neutralization of SARS-CoV-1, but partial cross-protection against endemic seasonal coronavirus like HCoV-OC43, -NL63, and −229E (Lawrenz et al., 2022).

Additionally, immunization with spike proteins before SARS-CoV-2 immunization impedes the generation of SARS-CoV-2 neutralizing antibodies in mice, thus, they amplify cross-reactive antibodies that are non-neutralizing antibodies (Lin et al., 2022). Inconsistencies in humoral immunity response is that prior studies did not examine the level of human common coronaviruses antibodies in the same individual before and after SARS-CoV-2 infection.

In connection to cell mediated immunity, a cross-reactive T cell response to SARS-CoV-2 and HCoV-NL63 and OC43 was demonstrated in a cohort of convalescent patients. However, they also displayed an increased number of CD4+ T cells than unexposed group throughout the 9-month study period (Wirsching et al., 2022).

Beyond the cross-protection between coronaviruses, other studies reported a decrease on the antibody (IgG, and IgA) levels to respiratory syncytial virus and influenza virus was reported during COVID-19 lockdowns (Grobben et al., 2022). In this study, decreased antibody levels to common respiratory viruses in human milk was also observed during COVID-19 pandemic, which translated in lower passive immunity in children.

## 9 General conclusions

This article constitutes a comprehensive review on the infection mechanism and replication of HCoV-NL63, compiling the current HCoV-NL63-related research related to virus entry and replication mechanism, including virus attachment, endocytosis, genome translation, and replication and transcription, in comparison with other coronaviruses. Moreover, cumulative knowledge on the susceptibility of different cells to HCoV-NL63 infection *in vitro*, was also reviewed, which is essential for successful virus isolation and propagation, and contribute to address different scientific questions from basic science to the development and assessment of diagnostic tools, and antiviral therapies. Finally, this review discussed different antiviral strategies that have been explored to suppress replication of HCoV-NL63, and other related human coronaviruses, by either targeting the virus or enhancing host antiviral mechanisms.

## Funding

NA.

## Declaration of Competing Interest

Authors declared no potential conflicts of interest with respect to the research, authorship, and/or publication of this article.

## Data Availability

Data will be made available on request.
